# Molecular Evaluation of Joubert Syndrome and Hearing Impairment in a Patient with Ataxic Cerebral Palsy

**DOI:** 10.1055/s-0043-1771184

**Published:** 2023-07-17

**Authors:** N. Sreedevi, N. Swapna, Santosh Maruthy, T. Jayakumar, Charles Sylvester

**Affiliations:** 1 Department of Speech-Language Sciences, All India Institute of Speech and Hearing, Mysore, India; 2 Department of Speech-Language Pathology, All India Institute of Speech and Hearing, Mysore, India; 3 Unit for Human Genetics, All India Institute of Speech and Hearing, Mysore, India

**Keywords:** Joubert syndrome, hearing loss, AHI1, GJB2

## Abstract

Joubert syndrome (JBTS) is a rare autosomal recessive or X-linked congenital brain malformation with strong genetic heterogeneity. Other neurological features of JBTS include hypotonia, ataxia, developmental delay, and cognitive impairment. Hearing loss with JBTS has been reported in the literature. We present the case of a 3.5-year-old boy born to a healthy consanguineous South Indian couple who was presented with ataxic cerebral palsy (CP) and hearing impairment; medical reports confirmed typical brain malformations of JBTS. Hearing impairment was screened by audiological assessment, which confirmed the presence of severe-profound hearing loss with outer hair cell dysfunction. Whole-exome sequencing (WES) was performed to know the molecular aspects of the condition and to detect any novel mutations. The homozygous mutation *AHI1* c.2023G > A associated with JBTS type 3 and *GJB2* c.71G > A mutation associated with hearing impairment were identified. Sanger sequencing was performed to validate the result and it identified heterozygous *AHI1* c.2023G > A and *GJB2* c.71G > A in the patient's parents. This study confirms the diagnosis of JBTS by WES helps identify the genetic causes of hereditary disorders that accelerate genetic evaluation and counseling for at-risk families.

## Introduction

Joubert syndrome (JBTS) is a rare neurodevelopmental ciliopathy characterized by cerebellar and brainstem malformation, hypotonia, respiratory deficit, ataxia, developmental delay, and cognitive impairment with congenital onset. 1 2 Axial brain imaging of JBTS is a characteristic malformation that resembles a molar tooth sign. 3 Along with the neurological aspects, over two-thirds of individuals with JBTS have organ defects such as ocular dystrophy, renal disease, hepatic fibrosis, and skeletal changes, which are noticeable at different ages with varied severity. 4 5 JBTS and related disorders are associated with a high prevalence of strabismus. 6 The epidemiology of JBTS is estimated between 1/80,000 and 1/100,000 live births. 7 JBTS is an autosomal recessively or X-linked inherited syndrome with a strong genetic heterogeneity and consanguinity that has been frequently associated with JBTS. 2 8 Hearing loss with JBTS has been reported in the literature. 9 10 The significant phenotypic overlap and wide variability of the ciliopathy can be explained by molecular and cellular etiology. 11 To identify the underlying genetic defects in the proband, we performed whole-exome sequencing followed by direct sequencing. In this study, we show that mutations in *AHI1* , which encodes the Jouberin protein at the JBTS3 locus, cause JBTS and *GJB2* , which encodes Connexin 26 protein, causes deafness in a south Indian patient.

## Clinical Report and Molecular Analysis

The proband, a 3.5-year-old boy, was born to a healthy consanguineous couple. He was born full term and was delivered through cesarean section weighing 3,500 g, with no birth asphyxia. He was hypotonic as an infant and presented with ataxia and global developmental delay. Delayed speech and language and severe mental retardation were noted ( Table 1 ). Audiological evaluation revealed severe to profound hearing loss; distortion-product otoacoustic emission (DPOAE) was absent in both ears. Magnetic resonance imaging (MRI) has confirmed JBTS as per previous medical records. Blood samples were collected from the patient and his family members after obtaining written informed consent. Genomic DNA was extracted from peripheral blood by using PureLink Genomic DNA Mini Kit (Thermo Fisher Scientific, United States) according to the manufacturer's instructions. Whole-exome sequencing was performed for the proband. The exome libraries were constructed using the Ion AmpliSeq Exome RDY kit (Thermo Fisher Scientific, United States) and sequenced on the Ion Proton sequencing platform (Life Technologies, United States). Variants were called using the Torrent Variant Caller plug-in using the software console of the Torrent server. Variants were annotated by Ion Reporter (Thermo Fisher Scientific, United States) using the human reference genome (hg19). Sanger sequencing was performed for variant validation of the proband and the proband's parents' samples by amplifying the *AHI1* loci using the primers (forward: 5′-TTAATAACCCCTAACCCCATCTC-3′, reverse: 5′-TTTCTCTGTGCTGCAAATGTCT3′) and the *GJB2* loci were amplified using the primers (5′-TCTTTTCCAGAGCAAACCGA-3′, reverse: 5′-GACACGAAGATCAGCTGCAG-3′) for a total volume of 10 μL. An initial denaturation step at 95°C for 2 minutes was followed by 35 cycles of 98°C for 25 seconds, 65°C for the *AHI1* variant, and 60.3°C for the *GJB2* variant for 45 seconds for annealing, 72°C for 30 seconds for elongation, and final extension at 72°C for 7 minutes. The polymerase chain reaction (PCR) products were evaluated using a 2% agarose gel electrophoresis. PCR products were labeled with BigDye Terminator v3.1 Cycle Sequencing Kit (Applied Biosystems, United States). The above-mentioned PCR primers ( *AHI1* forward and *GJB2* forward) were used as sequencing primers and then analyzed by ABI 3500 Genetic Analyzer (Applied Biosystems, United States). Sequence data were analyzed with SeqScape v3 software (Applied Biosystems, United States).

**Table 1:** Clinical features of the patient with mutations in *AHI1* and *GJB2* gene

ID	CP_69A
Sex	Male
Age	3.5 y
National origin	India
Variant 1	*AHI1* c.2023G > A p.D675N
Variant 2	*GJB2* c.71G > A p.W24X
Molar tooth sign	+
Hypotonia	+
Ataxia	+
Developmental delay	+
Mental retardation	+
Strabismus	+
Delayed speech and language	+
Hearing loss	+
Renal involvement	NA
Respiratory abnormalities	NA
Liver involvement	NA
Limb anomalies	NA

Abbreviations: +, present; NA, information not available.

## Discussion

**Fig. 1:**
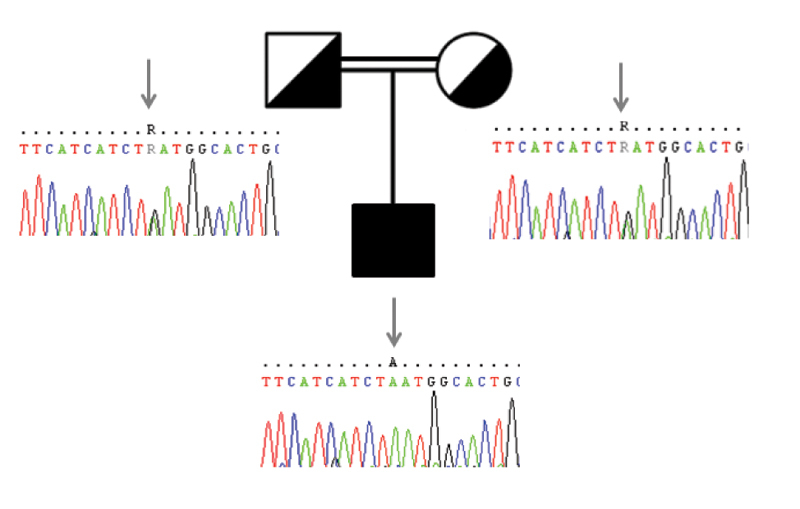
Pedigree of the family with genotypes of the variant *AHI1* c.2023G > A shown in corresponding pedigree members. Parents are heterozygous for the mutation.

**Fig. 2:**
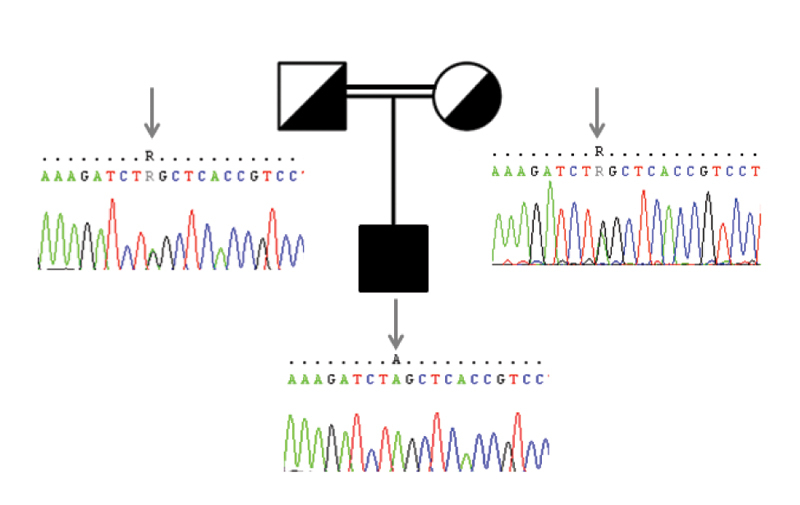
Pedigree of the family with genotypes of the variant *GJB2* c.71G > A shown in corresponding pedigree members. Parents are heterozygous for the mutation.

We present mutations in *AHI1* and *GJB2* genes in a patient clinically diagnosed with JBTS and hearing impairment. The *AHI1* variant (NM_001134831.2) c.2023G > A p.D675N and the *GJB2* variant (NM_004004.6) c.71G > A p.W24X, consistent with the clinical findings of Joubert syndrome 3 (JBTS3) and hearing impairment, respectively, were identified. The mutation *AHI1* c.2023G > A p.D675N is a classical feature of JBTS3 (OMIM #608629). 4 12 Heterozygous *AHI1* c.2023G > A p.D675N and heterozygous *GJB2* c.71G > A p.W24X were detected in the mother and father of the proband, which revealed the autosomal recessive mode of inheritance ( Figs. 1 and 2 ). The *AHI1* gene is located on chromosome 6q23.3, has 31 exons, and encodes Jouberin, a protein in the primary cilium with 1,196 amino acid residues containing a coiled-coil region, 7 WD40-repeats domain, and 1 SH3 domain, which is strongly expressed in the embryonic hindbrain and forebrain, which are required for both cerebellar and cortical development in humans. 13 The mutation *GJB2* c.71G > A p.W24X, which is the most common cause of severe to profound hearing impairment/autosomal recessive deafness (OMIM # 220290) in many populations, is also being reported in subjects with auditory neuropathy spectrum disorder. 14 The *GJB2* gene is located on chromosome 13q12.11, has two exons, and encodes connexin 26, a cochlear gap junction protein; mutations lead to an altered or loss of function of connexin 26, which appears to disrupt the assembly or function of gap junctions that alter the levels of potassium ions, which may damage the cells that are required for hearing. 15 16 Additional neurological features of JBTS and related disorders such as hypotonia, ataxia, developmental delay, mental retardation, and strabismus were also observed in the proband; no renal or liver involvement or no skeletal or respiratory anomalies were displayed ( Table 1 .)

The *AHI1* c.2023G > A p.D675N mutation has been previously reported only once in the literature. 4 To the best of our knowledge, this study is the second report of *AHI1* c.2023G > A associated JBTS and the first in India that is linked with *GJB2* c.71G > A associated hearing impairment. The only case report with sensorineural deafness in JBTS has also been reported from India. 9 Some patients with *GJB2* mutations have minimal or missing DPOAE. 17 Reduced DPOAE is a sign of nonfunctional outer hair cells (OHCs); OHCs are known to play a crucial role in the cochlear active process by electromotility. 18 Reduced electromotility of OHCs is linked with *GJB2* -related deafness; mutations in gap junctions can lead to the apoptosis of hair cells. 19 The presence of mild sensorineural hearing loss (SNHL) was also reported in three Dutch individuals with JBTS. 10 *AHI1* and other associated genes are linked with human brain development; changes in *AHI1* between hominids suggest a positive evolutionary selection in the lineage leading to humans. 12 *AHI1* is vital for axonal pathfinding mechanisms from the cortex to the spinal cord and mutations in *AHI1* cause neurological diseases 20 ; *AHI1* is also essential for synchronized movements of the hands and feet and might have been involved in the evolution of gait, which is distinctive of humans. 21 Since the proband presented in this study has ataxia, he might be labeled as a case of ataxic cerebral palsy (CP). However, studies have shown that the diagnosis of JBTS can be difficult as the presentation can be similar to cases of CP, 22 and JBTS can mimic ataxic CP in early life. 23 Cases of JBTS labeled as hypotonic CP were previously reported. 24 25 Similar to our case, molecular screening confirmed the presence of *AHI1* c.2023G > A p.D675N mutation that is associated with JBTS3, which prevents the misleading presentation.

To conclude, this study presented the molecular etiology of JBTS and hearing loss in a patient by detecting the disease-causing mutations *AHI1* c.2023G > A and *GJB2* c.71G > A of JBTS3 and hearing impairment, respectively. Despite its low incidence, JBTS should be considered a differential diagnosis that can accelerate the genetic evaluation process and make an informed decision for at-risk families for accurate genetic evaluation and counseling.
